# Supplementary material for: Multicycle Performance of CaTiO3 Decorated CaO-Based CO2 Adsorbent Prepared by a Versatile Aerosol Assisted Self-Assembly Method
Source: Nanomaterials (Basel). 2021 Nov 24;11(12):3188. doi: 10.3390/nano11123188 (PMC8703767; doi:10.3390/nano11123188)
Supplement: Supplementary file 1 [file nanomaterials-11-03188-s001.zip › nanomaterials-1447970-supplementary.pdf]

nanomaterials

Supplementary Materials

Multicycle Performance of $CaTiO_3$ Decorated CaO-based CO_2 Adsorbent Prepared by a Versatile Aerosol Assisted Self-Assembly Method

Ren Wei Chang [1,2], Chin Jung Lin [3,*] , and Ya Hsuan Liou [1,2,*]

[1] Department of Geosciences, National Taiwan University, Taipei 106, Taiwan; d06224005@ntu.edu.tw
[2] Research Center for Future Earth, National Taiwan University, Taipei 106, Taiwan
[3] Department of Environmental Engineering, National Ilan University, I-Lan 260, Taiwan
* Correspondence: lincj@niu.edu.tw (C.J.L.); yhliou@ntu.edu.tw (Y.H.L.)

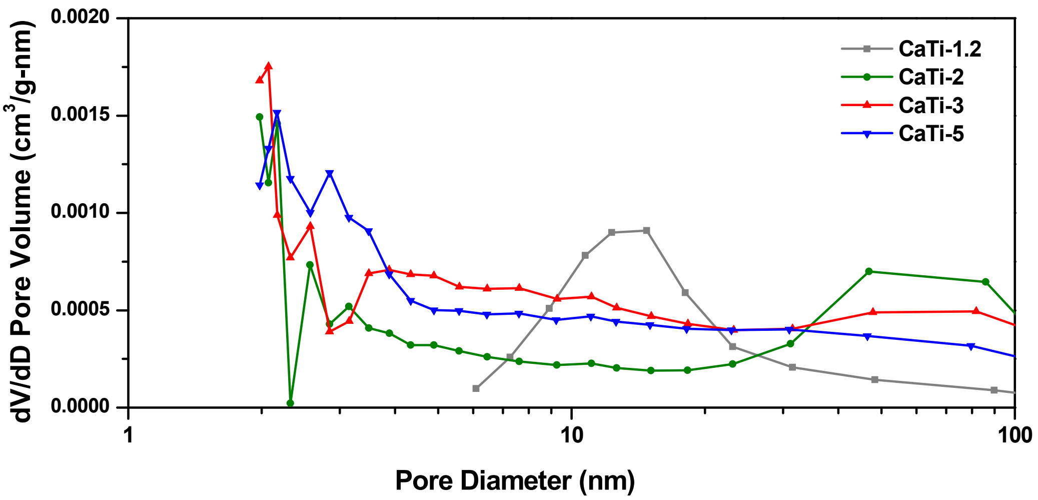

Figure S1. BJH pore size distribution result of calcium-based sorbents.
